# Immunemodulatory Effects of 5-Azacitidin Through Expansion of Functional Regulatory T Cells on Paraneoplastic Inflammation Associated With Myelodysplastic Syndromes: A Case Report

**DOI:** 10.3389/fonc.2018.00204

**Published:** 2018-06-05

**Authors:** Kentaro Serizawa, Hirokazu Tanaka, Yasuyoshi Morita, Takahide Taniguchi, Takashi Ashida, Itaru Matsumura

**Affiliations:** Department of Hematology and Rheumatology, Faculty of Medicine, Kindai University, Osakasayama, Osaka, Japan

**Keywords:** myelodysplastic syndrome, sweet’s syndrome, azacitidine, regulatory T cell, case report

## Abstract

Myelodysplastic syndrome (MDS) is a heterogeneous group of clonal disorders of hematopoietic stem cells, characterized by dysplastic hematopoiesis and dysregulated immune system resulting in various clinical conditions. Paraneoplastic inflammatory syndromes, which are well known to be associated with MDS, show response to immune-modulated therapy and often disappear during the course of hematologic management. Azacitidine (5-Aza) was shown to prolong survival of high-risk MDS patients, however, the effects of 5-Aza on paraneoplastic inflammation in MDS have yet to be elucidated. 5-Aza was administered to a 60-year-old man with MDS accompanying Sweet’s syndrome at a dose of 75 mg/m^2^/daily subcutaneously for 7 days every 28 days. 5-Aza was not only effective in controlling systemic symptoms caused by paraneoplastic inflammation, but hematologic improvements were also observed after four cycles of the 5-Aza treatment. Immune profiling in peripheral blood before and after 5-Aza treatment revealed that the effector and naive regulatory T cells in lymphocytes drastically increased after the 5-Aza treatment, i.e., 5-Aza might induce a shift in lymphocytic populations toward immunosuppression in this patient. Our results raised the immune-mediated effect of 5-Aza on both dysplastic hematopoiesis and paraneoplastic inflammation in myelodyplastic syndromes.

## Introduction

Myelodysplastic syndrome (MDS) is an acquired blood disorder characterized by varying degrees of ineffective hematopoiesis. Immunological abnormalities are an accepted fact in patients with MDS, which has been proposed to be implicated in disease initiation and progression (1). Paraneoplastic inflammatory syndromes are well known to be associated with MDS and their onset can precede, follow, or appear concurrent with the diagnosis of MDS (2–5). In general, paraneoplastic inflammatory disorders are less responsive to therapy than non-paraneoplastic cases, and standard treatment of the underlying MDS rarely improves symptoms (6, 7). DNA methyltransferase inhibitors, azacitidine (5-Aza), have been shown to increase the survival of patients with high-risk MDS (8). However, the effects of 5-Aza on paraneoplastic inflammation in MDS have yet to be elucidated. Herein, we describe the successful use of 5-Aza in a patient with MDS accompanying Sweet’s syndrome.

## Case Report: Clinical Presentation

A 60-year-old Japanese man suffered from a persistent high fever and progressive painful skin rash in the region of arms, lower abdomen, and legs. Laboratory work up revealed systemic inflammation and macrocytic anemia. The patient was referred to our department due to suspected systemic vasculitis.

An initial peripheral blood (PB) examination revealed anemia: hemoglobin 7.8 g/dL, platelets (Plt) 86 × 10^9^/L, and white blood cells 2.90 × 10^9^/L with 1.3% blasts, 0.3% metamyelocyte, 6% band forms, 55.7% segmented neutrophils, 4% monocytes, 0.7% eosinophils, and 31.7% lymphocytes. Laboratory tests also revealed an elevated C-reactive-protein level of 10.6 mg/dL and elevated liver enzyme levels (GOT 107 IU/L; GPT 79 IU/L; LDH 234 IU/L). The WT1 mRNA expression levels were increased (2.7 × 10^3^ copies/μgRNA). Extended serologic studies for autoimmune disease, and imaging studies for the detection of vasculitis failed to reveal the cause of the systemic inflammatory response syndrome.

**Figure 1:**
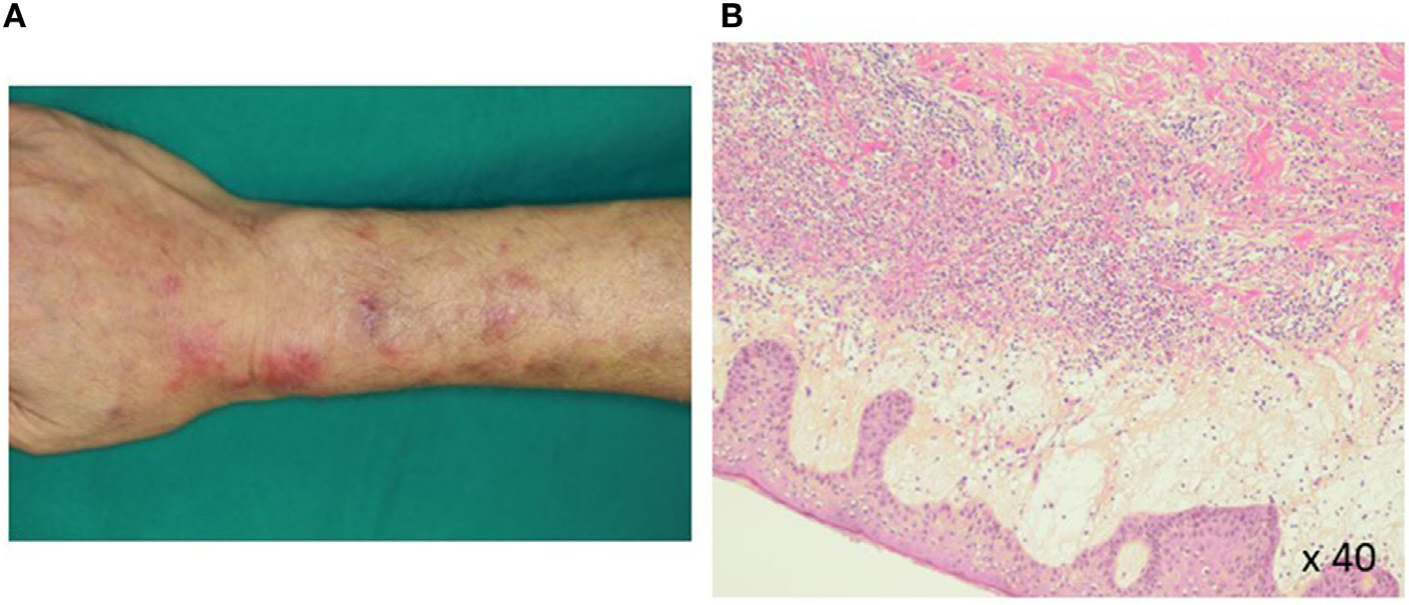
Histopathology of a Sweet’s syndrome lesion. Closer views **(A)** of Sweet’s syndrome lesions located on the upper arms are shown. The biopsy specimen **(B)** shows a confluent neutrophilic infiltrate in the reticular dermis and edema in the papillary dermis (hematoxylin and eosin staining).

**Figure 2:**
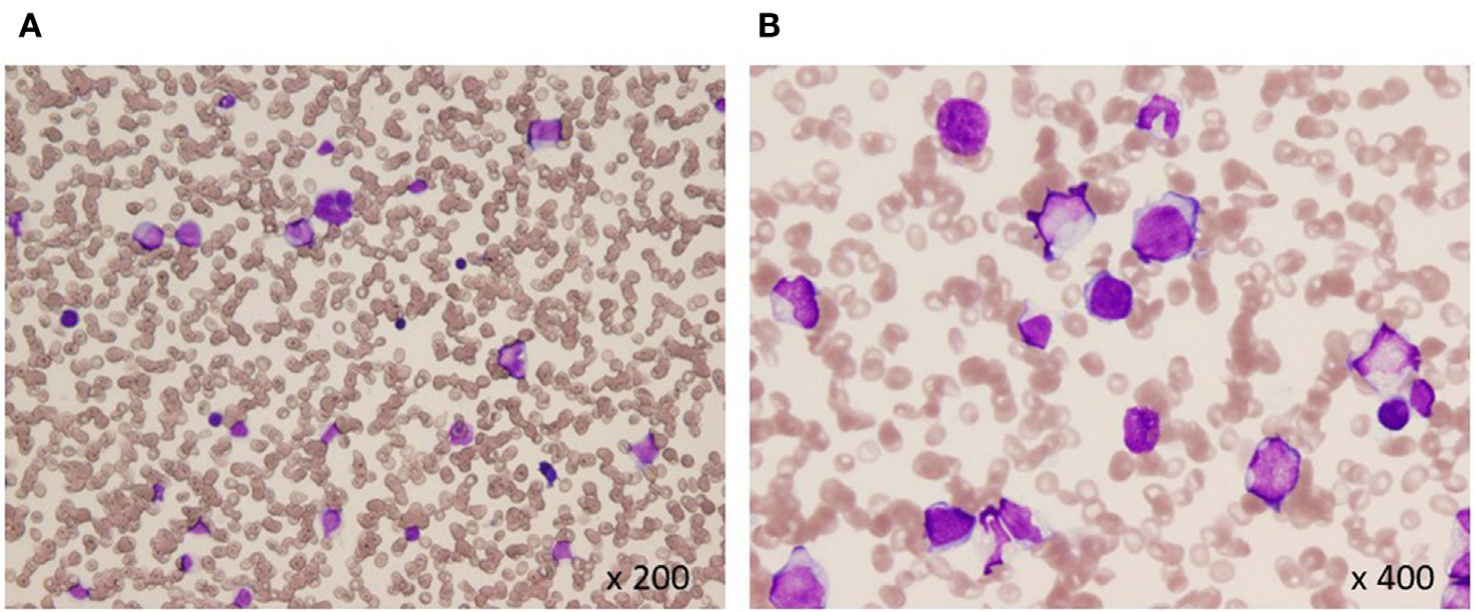
Bone marrow aspiration at diagnosis **(A,B)**. Hypocellular bone marrow with 14% blasts and dysplasia in all cell lineages (May-Giemsa).

Pathohistological work up of skin lesions revealed papillary dermal edema, swollen endothelial cells, and a diffuse infiltrate of predominantly neutrophils with leukocytoclasia (Figure 1). The overall picture was consistent with Sweet’s syndrome. Due to persistent cytopenia, bone marrow aspiration was also performed, showing 14.2% blasts with dysplasia in all cell lineages (Figure 2). MDS type refractory anemia with excess of blasts II was diagnosed. Metaphase banding analysis revealed complex cytogenetic including −5 and −7. The patient was, therefore, scored intermediate-2 risk according to the International prognostic scoring system. Taken together, Sweet’s syndrome occurred in a paraneoplastic form. After four courses of a bridging therapy with 5-Aza at standard dosage (75 mg/qm; q7 days; 4-weekly), allogeneic stem cell transplantation was performed. Four cycles of the 5-Aza treatment lead to steady disappearance of cutaneous lesions and allowed remarkable improvement of patient’s general condition and hematologic improvements were also observed according to the International Working Group 2006 criteria after four cycles of the 5-Aza treatment. Furthermore, the treatment of 5-Aza markedly decreased and normalized the WT1 mRNA expression levels (1.3 × 10^2^ copies/μgRNA).

**Figure 3:**
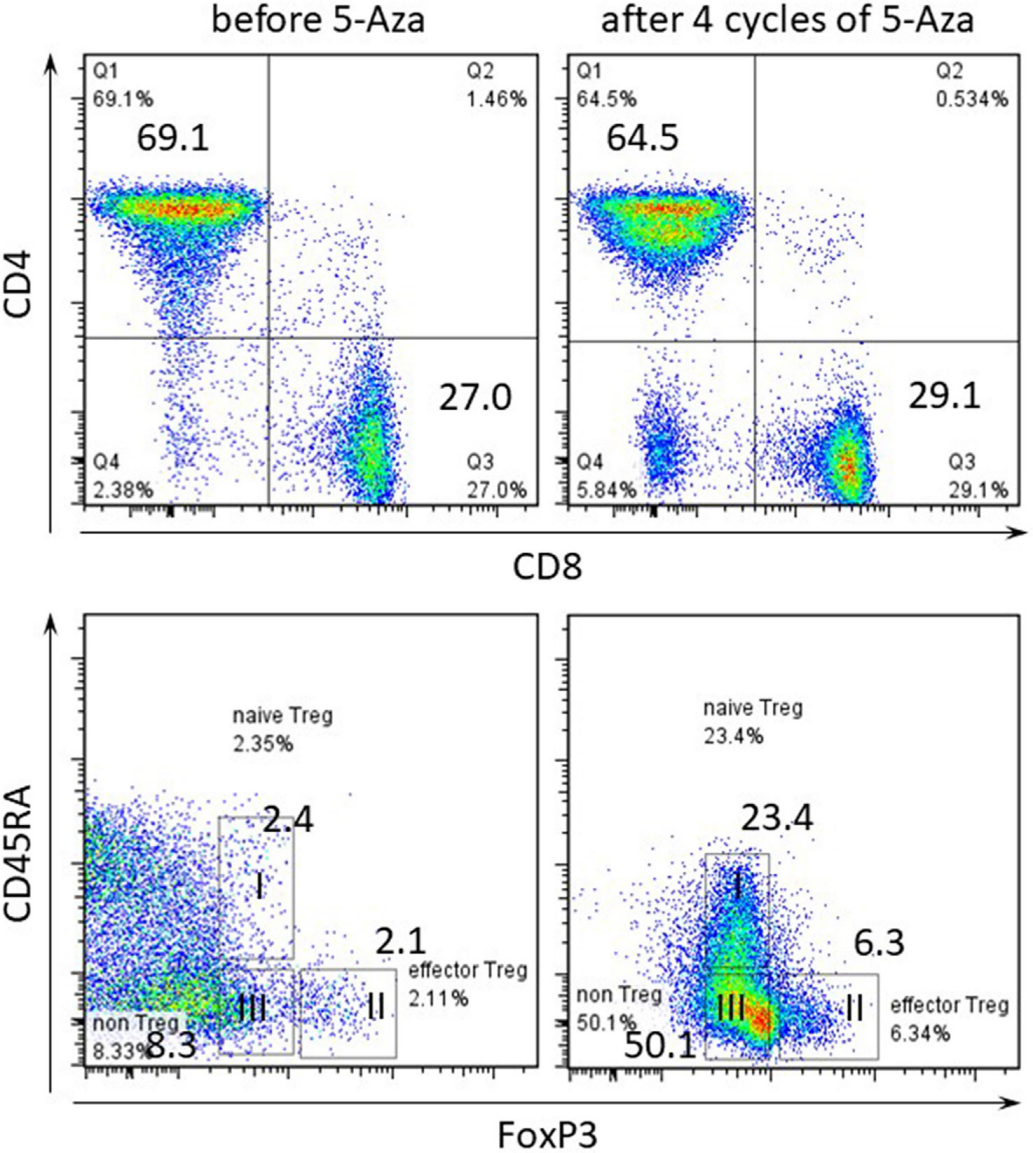
Frequency of regulatory T cells within CD4-positive T cell compartment in peripheral blood (PB). Flow cytometry of PB gated on CD3-positive in upper panel and CD3 CD4-double positive T cells in lower panel. Numbers indicate percentage in each quadrant. I CD4+CD45RA+forkhead box p3 (FoxP3) low naïve Treg, II CD45RA-FoxP3 high effector Treg, III CD45RA-FoxP3 low non-Treg.

Then, we performed immune profiling in the bone marrow of patients before and after 5-Aza therapy for four cycles. At least 1 × 10^6^ PB was initially labeled for dead cells with 7-AAD (Becton-Dickinson, San Jose, CA, USA) and antibodies against surface antigens, anti-CD8 Alexafluor 700, anti-CD4 APC, anti-CD45RA PECy7 (all from Becton-Dickinson), was used. For regulatory T cells (Treg cells) staining we used the anti-human forkhead box p3 (FOXP3) BV421 conjugate after fixation and permeabilization according to manufacturer’s instructions (Becton-Dickinson). Therapy with 5-Aza led to no significant increase in the absolute lymphocyte count with changes in the distribution of the CD4-positive T cells within the entire compartment (Figure 3). In parallel, the frequency of FoxP3-positive cells drastically increased in the PB during treatment. Especially, 5-Aza treatment led to an increase in the frequency of naïve and effector Treg cells (Fraction I and II, respectively, Figure 3).

## Discussion

The malignancy-associated Sweet’s syndrome can occur as a paraneoplastic syndrome in patients with MDS (6). Systemic corticosteroids are the therapeutic gold standard for Sweet’s syndrome, whereas, paraneoplastic inflammatory disorders are less responsive to therapy than non-paraneoplastic cases (6, 7). Recently, the efficacy of 5-Aza treatment in Sweet’s syndrome associated with MDS has been described in several reports (9, 10). However, a number of studies have pointed to important immunoregulatory effects of 5-Aza but the mechanisms are not fully understood. Several reports suggested that 5-Aza may expand Treg associated with increased FOXP3 expression due to FOXP3 promoter demethylation (11, 12). FOXP3 is a key transcription factor for the development and function of natural CD4+ Treg cells. Also, it has been reported that FOXP3 is easily induced when T cells continue to be stimulated with 5-Aza *in vitro* (12). This report evidences that since FOXP3 was induced by 5-Aza, the FOXP3-positive fraction increased in our case. Furthermore, human FoxP3+CD4+ T cells were reported to compose of three phenotypically and functionally distinct subpopulations: CD45RA+FoxP3 low resting naïve Treg cells and CD45RA−FoxP3 high functional and effector Treg cells, and cytokine-secreting CD45RA−FoxP3 low non-suppressive T cells (13). It was proved that naïve and effector Treg cells have T cell inhibitory effect by CFSE assay. Especially, its effect was remarkable with effector Treg. Therefore, the increase of these populations’ shows to enhance the immunosuppressive effect. The proportion of three subpopulations differed between patients with immunological diseases. In our case, the frequency of FoxP3-positive cells, especially, naïve and effector Treg cells drastically increased in the PB during 5-Aza treatment, i.e., 5-Aza might clinically induce a shift in lymphocytic populations toward immunosuppression.

5-Aza has been shown to improve both overall survival and quality of life in patients with high-risk MDS (8). Our patient also achieved improvement of PB counts, steady reduction of bone marrow blast count, and WT1 expression level after the 5-Aza treatment. Because, in the context of anti-tumor immune system, effects of 5-Aza in our case should be favorable for dysplastic clones leading to disease progression (14), another mechanisms of 5-Aza against MDS cells should be contributed.

## Concluding Remarks

In conclusion, paraneoplastic inflammatory syndromes are well known to be associated with MDS. We describe the case of a patient with MDS accompanying Sweet’s syndrome who successfully treated with 5-Aza. The dual role of 5-Aza in immune system for pathophysiology of MDS is challenging, and rigorous studies are needed to establish the value of immune modulation as a treatment of MDS with paraneoplastic syndromes.

## Consent

Written informed consent in accordance with the Declaration of Helsinki was obtained from patient for analysis, publication of this case report, and any accompanying images.

## Ethics Statement

This study was carried out in accordance with the recommendations of Kindai University ethical committee. The protocol was approved by the Kindai University ethical committee. All subjects gave written informed consent in accordance with the Declaration of Helsinki.

## Author Contributions

All authors read and approved the final manuscript. KS, YM, TT, and TA collected and provided data on the inpatient and outpatient treatment of the cases presented. KS, YM, and TT performed documentation of bone marrow aspirates. KS, HT, and IM analyzed data, compiled diagnostic data, and wrote the manuscript.

## Conflict of Interest Statement

This work was made possible by institutional funding. The authors declare that the research was conducted in the absence of any commercial or financial relationships that could be construed as a potential conflict of interest.
